# Computed Tomography Diagnostic of Uncommon Case of Osteopetrosis in 80-Year-Old Man—Case Report

**DOI:** 10.3390/medicina56100518

**Published:** 2020-10-02

**Authors:** Witold Krupski, Joanna Kruk-Bachonko, Marcin R. Tatara

**Affiliations:** 1 II Department of Radiology, Medical University in Lublin, ul. Staszica 16, 20-081 Lublin, Poland; krupskiw@wp.pl (W.K.); asiakruk1@wp.pl (J.K.-B.); 2 Department of Animal Physiology, Faculty of Veterinary Medicine, University of Life Sciences in Lublin, ul. Akademicka 12, 20-950 Lublin, Poland

**Keywords:** calcium hydroxyapatite (Ca-HA), densitometry, Hounsfield units (HU), osteopetrosis, quantitative computed tomography (QCT)

## Abstract

*Background and Objectives:* During osteopetrosis course, impaired bone remodeling induces skeletal osteosclerosis and abnormally dense bones, which, however, are brittle and susceptible to low-energy fractures. In this study, radiological evaluation and densitometric measurements of several bones of the skeleton in one of the oldest patients in the world suffering from osteopetrosis was presented. *Materials and Methods*: Volumetric bone mineral density measurements of the examined bones in an 80-year-old man were performed using two different quantitative computed tomography techniques. *Results:* The obtained results show higher values of the volumetric bone mineral density of the trabecular bone in lumbar spine than in the cortical bone compartment. T-score and Z-score in this patient reached values of 27–28 and 31–32, respectively. *Conclusions:* The obtained densitometric data may serve for further diagnostic purposes of osteopetrosis. As documented, the severity of the osteosclerotic changes of bones were higher in this patient than in most other described cases. Moreover, radiological signs diagnosed in this patient were characteristic for all types of osteopetrosis making this case very uncommon.

## 1. Introduction

Physiological skeletal growth, development, and bone tissue homeostasis maintenance require two essential processes such as bone modeling and remodeling. Bone modeling is essential for proper skeletal growth and it is associated with changes of bone size and shape and physiological adaptation to mechanical loading. Bone matrix protein synthesis, especially in terms of bone collagen fibers formation and maturation, is strictly associated with following bone tissue mineralization providing mechanical strength and stiffness of bones [1]. The remodeling process is responsible for physiological rebuilding of the developed bones due to bone tissue resorption and its following formation. Old, used, and damaged bone tissue is physiologically replaced by newly formed bone matrix in response to cellular signals [2]. To maintain the optimal amount of bone tissue in the skeleton, the physiological balance between bone resorption and following bone formation is critical for skeletal health. The domination of the bone resorption processes over formation rate of bone tissue leads to osteopenia and osteoporosis, while the opposite situation may result in excessive bone tissue accumulation in skeletal structures [2,3].

Osteopetrosis (marble bone disease) is a heterogeneous group of disorders characterized by defective osteoclastic bone resorption resulting in increased bone mineral density (BMD) in bones of the axial and peripheral skeleton. The observed increased BMD occurs as the result of decreased bone resorption rate by osteoclasts and it is combined with diffuse sclerosis of the spine and tubular bones and metaphyseal widening. Osteopetrosis with normal or increased osteoclast counts usually results from failure to develop a ruffled border, while in forms with decreased osteoclast counts, an abnormality in the molecular pathways involved in osteoclastogenesis has been suggested [4,5,6,7]. In most types of osteopetrosis, a normal or increased number of osteoclasts may be present; however, the osteoclasts have an impaired capacity to acidify the extracellular compartment of the bone tissue that is crucial for physiological dissolution of bone matrix. Some osteopetrosis types may be associated with abnormal osteoclasts differentiation, including mutation of genes encoding osteoclast RANK receptor and its ligand [5]. Despite the high density, the bones are brittle and susceptible to low-energy fractures [8]. The severity of osteopetrosis may differ and severe forms may even lead to stillbirth or early death [9]. The clinical symptoms of osteopetrosis include short stature, increased fracture rate, delayed tooth eruption, mental retardation, hydrocephalus, progressive neurodegeneration due to compression of nervous structures, pancytopenia and impaired hematopoiesis, immune dysfunction, skin abnormalities, renal tubular acidosis, hepatosplenomegaly, and hyperostosis [10]. Craniofacial bones, especially skull, are characterized by thickening of their structure that can be recognized using radiological techniques [11]. Osteopetrosis may be related to optic canal stenosis leading to partial or complete blindness due to optic nerve compression or obstruction of venous orbital outflow and papilledema [12]. The symptoms such as facial palsy, conductive hearing loss due to Eustachian tube encroachment, sensorineural hearing loss resulting from internal auditory canal narrowing, tonsillar herniation secondary to decreased intracranial volume, and meningoencephalocele may also occur as the result of osteopetrosis [11].

The aim of the study was to determine volumetric bone mineral density (vBMD) of the trabecular and cortical bone compartments in lumbar spine and other available parts of skeleton in 80-year-old patient suffering from osteopetrosis. Quantitative computed tomography (QCT) technique was used for the first time for densitometric evaluation of osteopetrosis in terms of vBMD of the spine, pelvis, cranium, mandible, and hyoid.

## 2. Materials and Methods

**Figure 1:**
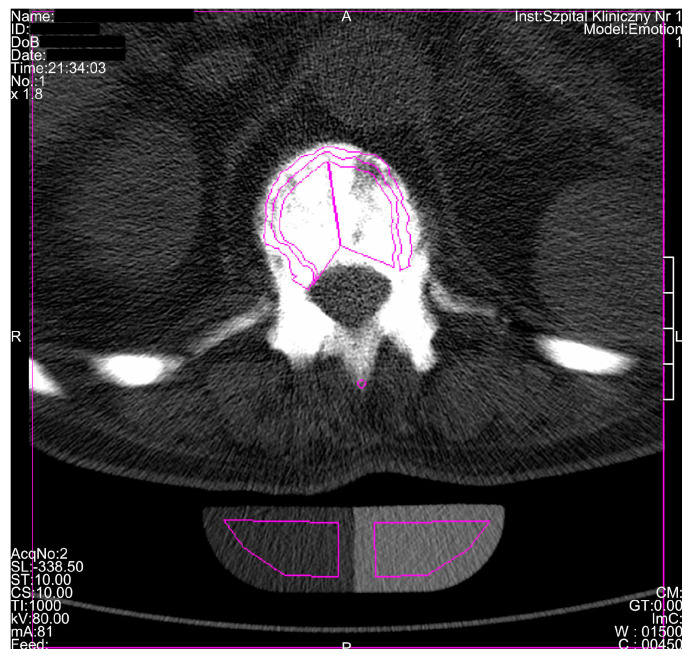
Volumetric bone mineral density (vBMD) measurement in the trabecular and cortical bone compartments of Th12 vertebrae using quantitative computed tomography (QCT) method and Osteo CT application package (Software Version B10/2004A), and expressed quantitatively as calcium hydroxyapatite (Ca-HA) density in the trabecular (TbCa-HA) and cortical bone (CbCa-HA). CT: the self-name of the software.

An eighty-year-old man was diagnosed in the II Department of Radiology of the Medical University in Lublin, Poland, using diagnostic imaging techniques due to a fall. Standard skull roentgenogram has shown no traumatic changes of bones but has revealed thickening of cranial bones and their osteosclerotisation typical for osteopetrosis. After that, the computed tomography (CT) examination of the skull and lumbar spine and quantitative computed tomography (QCT) evaluation were performed using spiral or sequential scanning procedure and Somatom Emotion apparatus equipped with Somaris/5 VB10B software (Siemens, Erlangen, Germany). Volumetric BMD (vBMD) was measured separately for the trabecular and the cortical bone of the vertebral body using 10-mm-thick, cross-sectional scans, positioned at 50% of each vertebral body length (Th12 and L1-L5). Both the trabecular and cortical vBMD were measured using Osteo CT application package (Osteo CT Software Version B10/2004A, Siemans, Erlangen, Germany) and expressed quantitatively as calcium hydroxyapatite (Ca-HA) density in the trabecular (Tb_Ca-HA_) and cortical bone (Cb_Ca-HA_) compartments (Figure 1).

**Figure 2:**
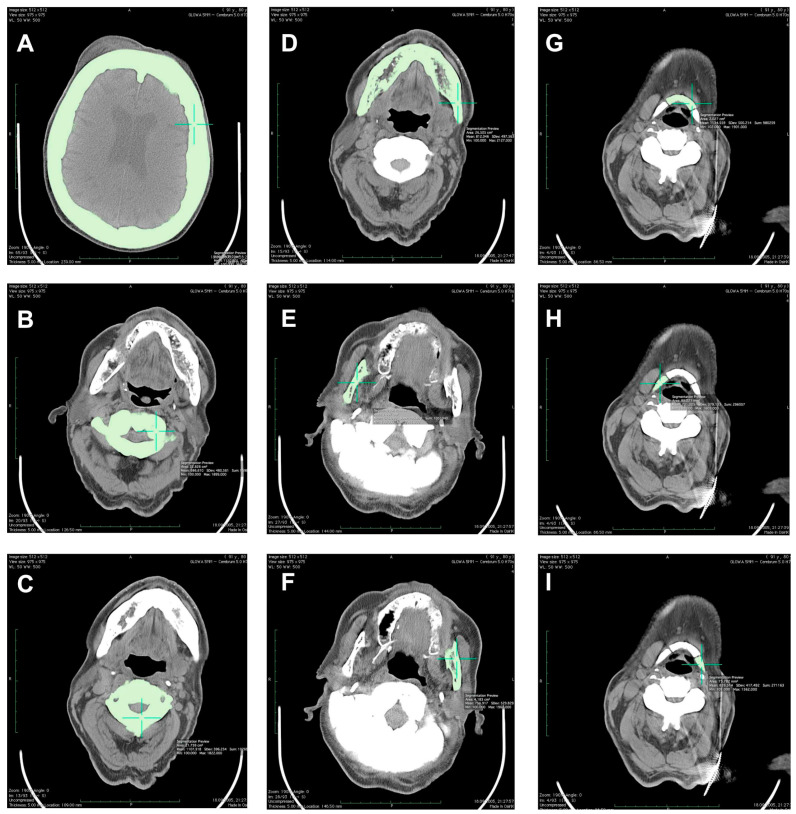
Measurement of volumetric bone mineral density (vBMD) of the cranium (**A**), first cervical vertebra—C1 (**B**), second cervical vertebra—C2 (**C**), mandibular body (**D**), right and left mandibular ramus (**E**,**F**), middle part of the hyoid (**G**), and right and left part of the hyoid (**H**,**I**) in g/cm^3^ using OsiriX software for a MacPro 29-ZRL computer. The value of the vBMD measurement was combined for the trabecular and cortical bone compartments. The volume-of-interest of the measuring scans was limited between the minimum and maximum density of the investigated bones at 100 and 3071 Hounsfield units.

The results of Ca-HA density measurements using Osteo CT software were expressed in mg of Ca-HA per milliliter (mg Ca-HA/mL). Moreover, T-score and Z-score values were determined for the examined patient. As defined previously, T-score value describes the number of standard deviations (SD) by which the BMD (or vBMD) in an individual differs from the mean value expected for young healthy sex-matched reference population. Z-score value describes the number of SD by which the BMD (or vBMD) in an individual differs from the mean value expected for age- and sex-matched reference population [13]. Patient scanning procedure was performed with the water-and bone-equivalent calibration phantom serving as a standard for such measurements [14]. Using OsiriX software for a MacPro 29-ZRL computer, the obtained digital data from CT scanning procedure were also analyzed to measure combined vBMD of the trabecular and cortical bone compartments in g/cm^3^. The volume-of-interest (VOI) of the measuring scans (3-mm thick for L3–L5 and pelvis or 5-mm thick for cranium, C1–C3, mandible, and hyoid) was limited between the minimum and maximum density of the investigated bones at 100 and 3071 Hounsfield units (HU), respectively (Figure 2). Using such a procedure, determination of the values of vBMD in the cervical (C1–C3) and lumbar spine (L3–L5) at 50% of the vertebral body length was performed. Moreover, vBMD of the cranium (measuring scan at the 50% of its height), mandible (alongside long axis of the mandibular body and separately for the right and left ramus), hyoid (separately for the middle, right, and left parts), as well as right and left parts of the pelvis (measuring scan at the line of 50% of L5 vertebral body length) was determined.

## 3. Results

**Figure 3:**
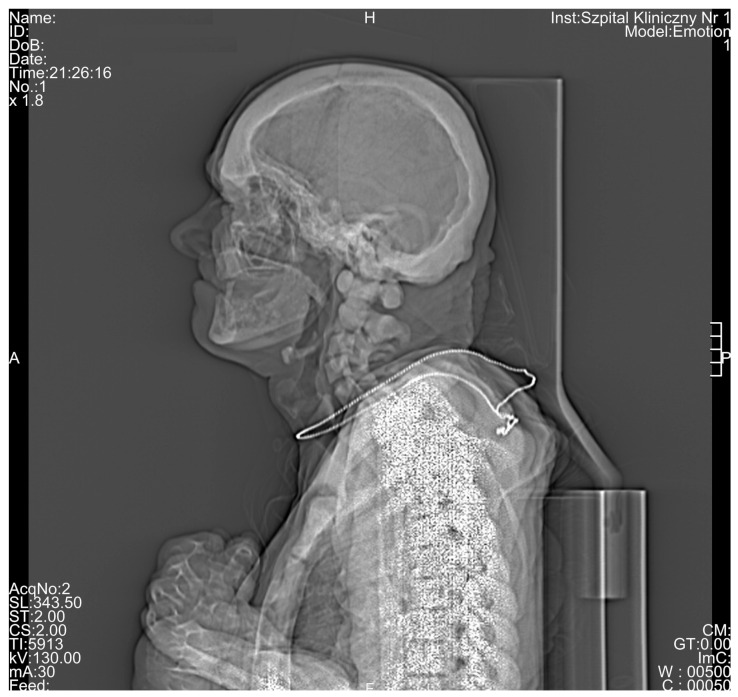
Scout view topogram obtained using computed tomography showing visible general osteosclerosis, especially within the skull base, the skull vault, and sternum.

**Figure 4:**
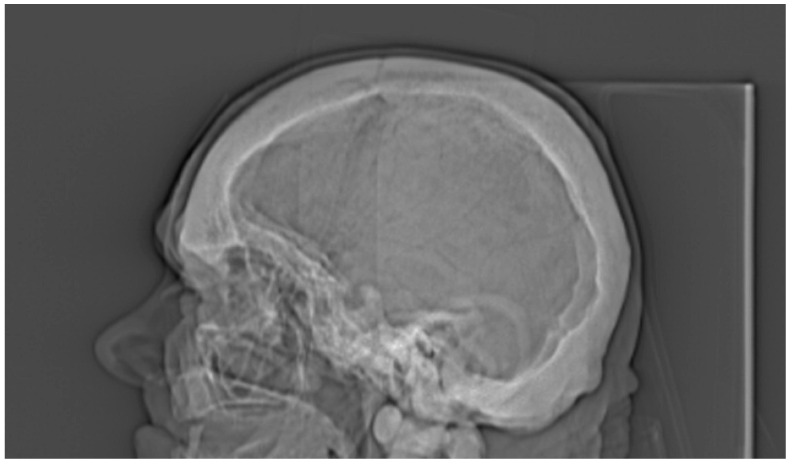
Frontal sinus aplasia or obliteration in 80-year-old patient suffering from osteopetrosis visible on scout view topogram of the cranium.

**Figure 5:**
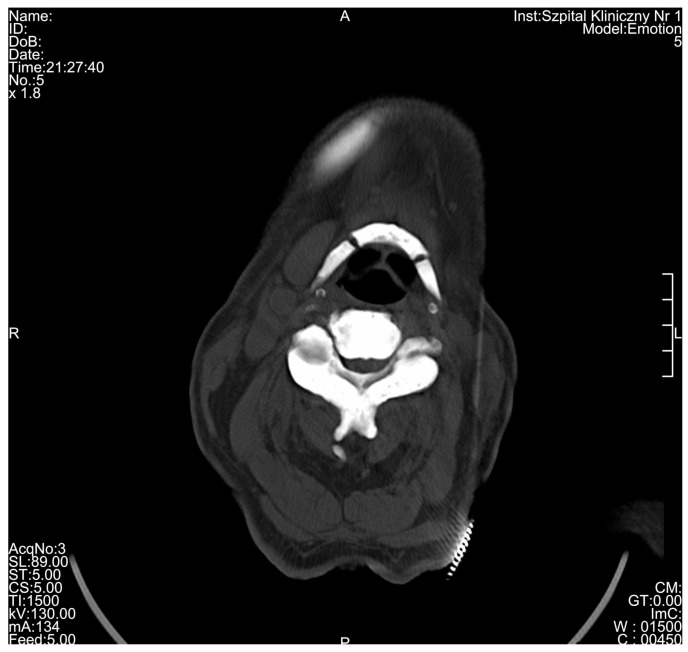
Significant osteosclerosis of the hyoid and cervical vertebra in 80-year-old patient suffering from osteopetrosis visible on cross-sectional computed tomography scan.

**Figure 6:**
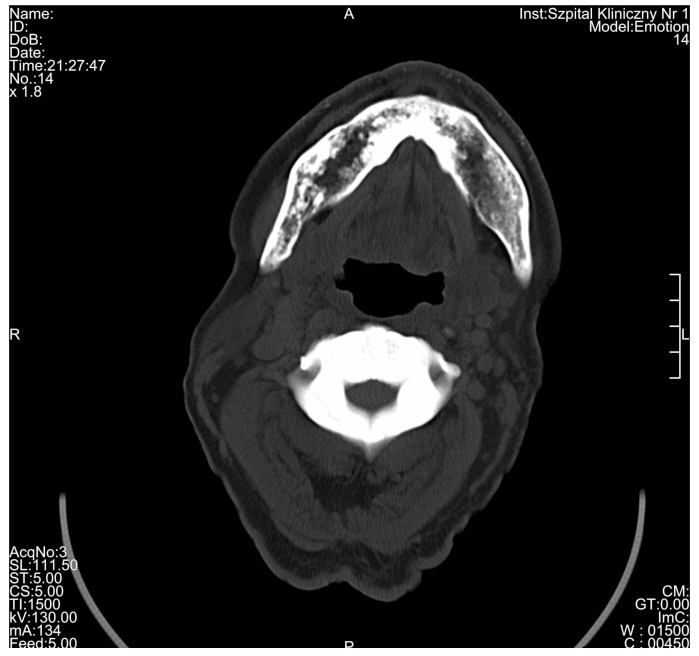
Significant osteosclerosis of the mandible and axis in 80-year-old patient suffering from osteopetrosis visible on cross-sectional computed tomography scan.

**Figure 7:**
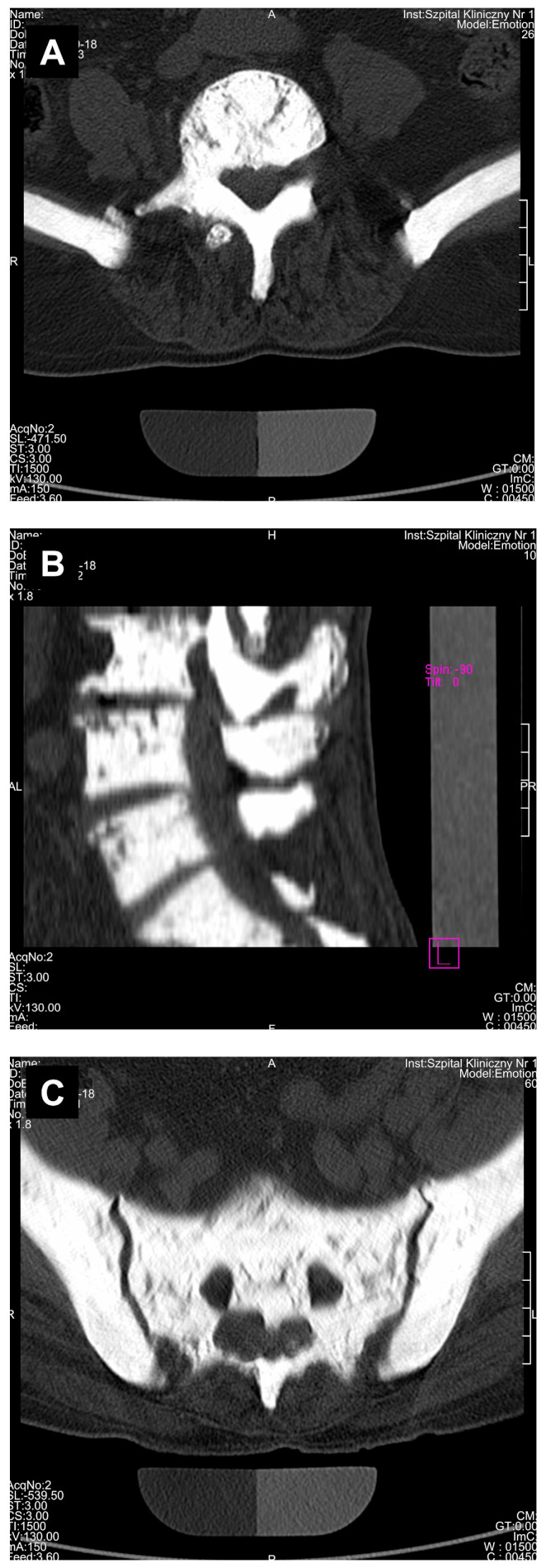
Osteosclerosis and marble view of the lumbar vertebrae (**A**,**B**), and pelvic (**A**,**C**) and sacral bones (**C**) in 80-year-old patient suffering from osteopetrosis visible on cross-sectional computed tomography reconstruction.

All the results of the densitometric measurements are shown in Table 1. Computed tomography diagnostic of the skull has shown increased total bone thickness reaching between 13 and 23 mm when measured on the skull cross-section positioned at 50% of its height (Figure 2A). The fully condensed osseous tissue (osteosclerosis) of the patient’s skull base and the skull vault was diagnosed using CT examination, and the diagnosed changes were visible both on the scout view topogram (Figure 3) and cross-sectional scans. Using CT method, bilateral auditory, optical, and facial nerve ducts’ stenosis, as well as frontal sinus aplasia or obliteration were diagnosed (Figure 4). Significant osteosclerosis of the hyoid and cervical vertebra (Figure 5), as well as mandible and axis (Figure 6) was revealed. Significantly increased skull bone thickness resulting in decreased cranial cavity volume was diagnosed (Figure 4). Moreover, significant visible vertebral osteosclerosis in CT examination of the lumbar and cervical spine and pelvic bones was found (Figure 5 and Figure 7).

## 4. Discussion

In this study, quantitative measurement of volumetric bone mineral density of the cervical and lumbar spine, cranium, mandible, hyoid, and pelvis was performed in an 80-year-old patient suffering from osteopetrosis. This case report provides for the first time densitometric results in such an age-advanced patient. Quantitative computed tomography was applied in this study to determine densitometric characteristic of the trabecular and cortical bone compartments, separately or combined. Except for unique reports on vBMD measurements in two adult patients (32-year-old woman and 44-year-old man) with the use of high resolution peripheral quantitative computed tomography (HR-pQCT) and in six children (age between 4.5 and 17.5 months) with the use of QCT, most of the other densitometric studies on osteopetrosis were performed using dual-energy X-ray absorptiometry (DEXA) [15,16,17]. In clinical practice, DEXA method is the most commonly utilized to BMD determination in patients [16]. In contrast to the DEXA method providing areal bone mineral density (aBMD) expressed in g/cm^2^, QCT and pQCT measure real volumetric BMD within separately defined VOI for the trabecular and cortical bone compartments. Moreover, vBMD may be determined for both of these compartments combined and expressed in g/cm^3^ or mg/cm^3^. A methodological advantage of QCT method for the determination of vBMD, in comparison to DEXA method, is a lack of potential measuring errors resulting from the presence of surrounding soft tissues, different size of bones, and osteoarthritic changes such as osteophytes frequently occurring in lumbar vertebrae [18]. In contrast to HR-pQCT or pQCT providing densitometric results from bones building only appendicular skeleton (forearm bones and tibia), QCT method allows additionally vBMD determination in bones of the axial skeleton such as different segments of spine, skull bones, and pelvis [17]. As shown in this study, contrary to DEXA and HR-pQCT, standard CT examination provides also qualitative and morphological evaluation of the examined bones. In most cases, both DEXA and QCT methods provide relevant comparison of the obtained results of the densitometric measurements to the healthy young and sex-matched population (T-score) or healthy sex- and age-matched population (Z-score) [13,15,19]. However, bone microarchitecture may be evaluated using HR-pQCT due to its relatively high resolution, reaching even 82 μm [17].

The obtained results in the current study have shown very high T-scores of the spine reaching values over 27 and 28. Z-score values were also extremely high reaching 31 and 32. The reference norm of vBMD measurement for 80-year person for the trabecular bone in lumbar spine was set at 71.8 mg Ca-HA/mL, while in this patient the physiological norm was exceeded by over 11–14 times, depending on particular spine vertebrae. The physiological norm of vBMD for the cortical bone compartment was not determined yet, unfortunately; however, it is surprising that in this study Cb_Ca-HA_ of all segments of the spine reached lower values than Tb_Ca-HA_ by 19–206 mg Ca-HA/mL. Both the measurements of Tb_Ca-HA_ and Cb_Ca-HA_ were based on the standard-containing calibration phantom provided by scanner producer. Independently on such calibration phantom, vBMD was also measured for several parts of the skeleton in HU and than converted to g/cm^3^. It was not possible to differentiate trabecular and cortical bone compartments determining separate ROIs and the obtained results concern both these combined compartments. As opposed to phantom-associated vBMD measurements where measuring scan was 10-mm thick, vBMD determination in g/cm^3^ was executed on 3-mm or 5-mm thick diagnostic scans, depending on examined skeletal site. Comparing both these densitometric methods of vBMD determination in the skeleton, result expression in g/cm^3^ seems to be more universal than in mg Ca-HA/mL since the measuring scale is the same for all available diagnostic equipment types including CT, HR-CT, pQCT, HR-pQCT, microCT, and nanoCT scanners [18,20,21,22]. However, there are no determined physiological ranges of vBMD, T-score, and Z-score for several bones of human skeleton. Volumetric BMD in g/cm^3^ may be determined from the diagnostic scans while the following measurements with the calibration phantom require an additional exposure of patient to X-rays when repeating the scanning procedure. On the other hand, phantom-based vBMD measurements for only densitometric purposes enable the limitation of X-ray exposure of the patient in comparison to the standard diagnostic scanning procedure. In such a case, vBMD determination in five lumbar vertebrae (L1–L5) requires totally only 5 cm of the spine exposure to X-rays, except for scout view topogram that must be executed in both these techniques. Densitometric measurements in g/cm^3^ may be performed for all diagnostically scanned bones while relatively short length of the calibration phantom limits its utilization to several segments of spine. As opposed to ex vivo experimental studies in animals, it is difficult in living patients to determine phantom-based vBMD of bones located in appendicular skeleton [23]. Moreover, there is no determined reference values of vBMD, T-score, and Z-score for the other parts of the skeleton such as femur and distal forearm.

The obtained results in this study are in accordance with data presented by Kaste et al. (2007) where Z-scores of L1 and L2 in children (4.5–17.5 months of life) suffering from infantile osteopetrosis reached values between 22.4 and 32.6. Volumetric BMD of the trabecular bone of spine has reached the values between 770.8 and 1029.3 mg/cm^3^ [15]. In a study using HR-pQCT method and describing 2 cases of osteopetrosis in adults (44-year-old male and 32-year-old female), average bone density (D100) reflecting total bone in the trabecular and cortical bone compartment was increased more than twice in comparison to normal subjects. Moreover, trabecular number, trabecular thickness, trabecular bone volume, and cortical thickness of the tibia and radius were increased [17]. In the other studies on 62 patients suffering from osteopetrosis and using DEXA method, data collected from 17–68-year-old patients were markedly increased showing mean Z-score values of lumbar spine, femoral neck, and total body of 9.84 (5.9–12.4), 10.14 (3.1–14.8), and 8.23 (5.2–11.9), respectively [19]. Thus, it is considered that densitometric diagnosis of osteopetrosis using DEXA method shows Z-score within the range of + 3 to + 15 and this range is lower than in case of QCT method where a + 32 Z-score may be exceeded [7,15,16].

Regardless of densitometric measurements in the current study, a unique radiological characteristic in the described patient was diagnosed. Based on CT examination, general osteosclerosis including cervical and lumbar spine osteosclerosis, skull vault osteosclerosis, skull base osteosclerosis, pelvis osteosclerosis, sacral bone osteosclerosis, sternum osteosclerosis, mandible osteosclerosis, and hyoid osteosclerosis was confirmed (Figure 1, Figure 2, Figure 3, Figure 4, Figure 5, Figure 6 and Figure 7). Moreover, it was possible to provide basic vBMD values for the pelvis, cranium, mandible, and hyoid that were not reported previously elsewhere and may serve for further diagnostic purposes. Considering the radiological features of bones visible in the performed CT examination, such as general osteosclerosis, skull vault osteosclerosis, skull base osteosclerosis, and sandwich vertebrae, it can be concluded that all the radiological signs characteristic for all types of autosomal dominant osteopetrosis (ADO I, ADO II, and ADO III; Table 2) in this patient were seen [24,25,26,27].

Except for sacral bone and tail vertebrae visible on scout view topogram, it was not possible to confirm bone within bone sign in long bones in the performed CT examination, since the ROIs were limited to cranium and lumbar part of the spine. However, the confirmed osteosclerosis of the pelvic bones suggest also the incidence of this radiological feature in the examined patient (Figure 7).

## 5. Conclusions

In conclusion, this study shows, for the first time, radiological evaluation and densitometric measurements of several bones of the skeleton in one of the oldest patient in the world suffering from osteopetrosis. Volumetric BMD measurements of the examined bones were performed using two different QCT techniques and may serve as reference data for osteopetrosis for further diagnostic purposes. As documented, the severity of the osteosclerotic changes of bones were one of the highest in this patient when compared to the other described cases in worldwide literature. Moreover, radiological signs diagnosed in this patient were characteristic for all types of osteopetrosis making this case very uncommon.

## Figures and Tables

**Table 1:** Volumetric bone mineral density (vBMD) measurements of the spine, pelvis, cranium, mandible, and hyoid in an 80-year-old male patient suffering from osteopetrosis.

Investigated Bone	Tb_Ca-HA_ (mg Ca-HA/mL)	Cb_Ca-HA_ (mg Ca-HA/mL)	T-Score	Z-Score	vBMD (g/cm^3^)
Spine					
Th_12_ vertebra	834.1	800.3	-	-	-
L_1_ vertebra	918.5	813.5	-	-	-
L_2_ vertebra	927.6	861.5	-	-	-
L_3_ vertebra	860.6	816.4	-	-	1.936
L_4_ vertebra	865.7	846.6	-	-	1.943
L_5_ vertebra	1039.8	833.7	-	-	1.912
Th_12_-L_2_ vertebrae	893.4	825.1	27.12	31.00	-
L_3_-L_5_ vertebrae	922.0	832.2	28.20	32.08	1.930
L_1_-L_5_ vertebrae	922.4	834.3	-	-	-
Reference value for 80-year-old man	71.8	-	-	-	-
C_1_ vertebra	-	-	-	-	1.987
C_2_ vertebra	-	-	-	-	2.107
C_3_ vertebra	-	-	-	-	2.010
Pelvis					
Right ilium	-	-	-	-	1.954
Left ilium	-	-	-	-	1.924
Cranium					
50% of cranium height	-	-	-	-	2.111
Mandible					
Mandibular body	-	-	-	-	1.812
Right ramus	-	-	-	-	1.678
Left ramus	-	-	-	-	1.757
Hyoid					
Middle part	-	-	-	-	2.135
Right part	-	-	-	-	1.731
Left part	-	-	-	-	1.840

“-” indicates lack of data.

**Table 2:** Autosomal dominant osteopetrosis (ADO) type classification according to radiological features *.

Radiological Feature	ADO Type I	ADO Type II	ADO Type III	Presented Case
General osteosclerosis	+	–	+	+
Skull vault osteosclerosis	+	–	+	+
Skull base osteosclerosis	–	+	–	+
Sandwich vertebrae (Ruger–Jersey spine)	–	+	–	+
Bone within bone	–	+	+	+
Hyoid sclerosis	–	–	–	+

“+” indicates presence of radiological feature. “–” indicates absence of radiological feature. * ADO type classification presented according to literature data [9,24,25,26,27].
